# Functional Role of Transient Receptor Potential Channels in Immune Cells and Epithelia

**DOI:** 10.3389/fimmu.2018.00174

**Published:** 2018-02-07

**Authors:** Mohammad Khalil, Korina Alliger, Carl Weidinger, Cansu Yerinde, Stefan Wirtz, Christoph Becker, Matthias Agop Engel

**Affiliations:** ^1^Universitätsklinikum Erlangen, Medizinische Klinik 1, Erlangen, Germany; ^2^Charité Universitätsmedizin Berlin, Campus Benjamin Franklin Medizinische Klinik für Gastroenterologie, Infektiologie und Rheumatologie, Berlin, Germany

**Keywords:** colitis, macrophages, cytokines, neuropeptides, neurogenic inflammation, nociception

## Abstract

Transient receptor potential (TRP) ion channels are widely expressed in several tissues throughout the mammalian organism. Originally, TRP channel physiology was focusing on its fundamental meaning in sensory neuronal function. Today, it is known that activation of several TRP ion channels in peptidergic neurons does not only result in neuropeptide release and consecutive neurogenic inflammation. Growing evidence demonstrates functional extra-neuronal TRP channel expression in immune and epithelial cells with important implications for mucosal immunology. TRP channels maintain intracellular calcium homeostasis to regulate various functions in the respective cells such as nociception, production and release of inflammatory mediators, phagocytosis, and cell migration. In this review, we provide an overview about TRP-mediated effects in immune and epithelial cells with an emphasis on mucosal immunology of the gut. Crosstalk between neurons, epithelial cells, and immune cells induced by activation of TRP channels orchestrates the immunologic response. Understanding of its molecular mechanisms paves the way to novel clinical approaches for the treatment of various inflammatory disorders including IBD.

## Introduction

The transient receptor potential (TRP) ion channel family consists of 28 members, which are divided into six subsets: TRPC (“canonical”), TRPM (“melastatin”), TRPV (“vanilloid”), TRPA (“ankyrin”), TRPML (“mucolipin”), and TRPP (or PKD) (“polycystin”) (1). TRP channels are membrane proteins with substantial cation permeability, preferentially high calcium ion permeability, and calcium signaling plays a central role in many physiological processes. TRP receptors are polymodal ion channels with an exceptional role in the integration of various environmental stimuli including mechanical, thermal, or chemical stimuli. Inhering this function they are likely to be sensors for monitoring specific responses to different exogenous and endogenous chemical noxious and physical stimuli. As such, various TRP channels play an essential role in somatic and visceral nociception (2, 3). Upon activation, TRP channels also control the release of immunomodulatory neuropeptides such as substance P (SP) and calcitonin gene-related peptide (CGRP), the so-called neurogenic inflammation. During recent years, increasing evidence has demonstrated an important role of many TRP channels outside the nervous system in the context of inflammation; findings that extend the role of TRP channels in the regulation of inflammation beyond neuropeptide release. To date, only little is known about the functional role of TRP channels in the immune system. Moreover, recent reports describe a fundamental role of TRP channels in epithelial cells in mediating cytokine/chemokine release as well (4). In summary, TRP channel activation induces immunomodulatory effects on multiple levels. This review will focus on the role of TRP channels in immune cells (with a focus on macrophages and T cells) and epithelial cells in general, with an additional special focus on TRPs in intestinal inflammation. Recognizing the large family of TRP channels, this mini-review focuses on TRPA1, TRPM8, TRPV1, and TRPV4 (alphabetical order), which are the most relevant TRP channels in the present context based on published literature until today. Table S1 (available in supplementary material) gives an overview of receptor expression and resulting biological effects in the different cellular compartments.

## TRP Channel Function—General Role in Immune Cells and Epithelia

### TRPA1

TRPA1 is an irritant receptor that belongs to the ankyrin subfamily and is highly co-expressed with TRPV1 in a subset of sensory neurons (5). Only very little is known to date about TRPA1 expression in immune cells. The role of TRPA1 in macrophages was recently investigated in the context of the pathogenesis of atherosclerosis. Oxidized low-density lipoprotein (oxLDL) and the prototypical TRPA1 agonist allyl isothiocyanate (pungent ingredient in garlic) induced calcium transients in bone marrow-derived macrophages *via* TRPA1. TRPA1 expression was found to be upregulated in macrophage foam cells in atherosclerotic aortas of apolipoprotein E-deficient (apoE^−/−^) mice. Treatment with a selective TRPA1 antagonist HC030031 (HC) led to aggravation of oxLDL-induced lipid accumulation and subsequently exacerbated atherosclerotic lesions in apoE^−/−^ mice. In addition, HC-treated apoE^−/−^ mice showed increased levels of serum HDL, triglycerides, total cholesterol, and the pro-inflammatory cytokines IL-1β, TNF-α, MCP-1, IL-6, and macrophage inflammatory protein-2 (MIP-2), which suggested a crucial anti-inflammatory role of TRPA1 in the pathogenesis of atherosclerosis and cholesterol metabolism of macrophage foam cells (6). Previously, another group provided evidence about the functional expression of TRPA1 in peritoneal macrophages. LPS-stimulated cannabichromene-treated (CBC, cannabinoid TRPA1 agonist) peritoneal macrophages showed a significantly decreased level of nitrite (stable metabolite of nitric oxide) compared to LPS-stimulated peritoneal macrophages without CBC pretreatment. Nitric oxide acts as an abundant pro-inflammatory mediator, which indicates anti-inflammatory effects of TRPA1 activation by CBC in peritoneal macrophages. Interestingly, the TRPA1 antagonists AP-18 and HC had almost the same inhibitory effect on the nitrite production as TRPA1 activation, which indicated that the effect of TRPA1 agonists was due to receptor activation and subsequent desensitization (7). The TRPA1 agonists acrolein and crotonaldehyde were able to excite the release of TNF-α and IL-8 (CXCL8, a potent neutrophil chemoattractant) from the human macrophage cell line U937, whereas acrolein induced release of IL-8 from the THP-1 macrophage cell line and from human alveolar macrophages. In addition, the lipid peroxidation product 4-hydroxy-2-nonenal (4-HNE), a mediator of oxidative stress and a TRPA1 agonist was found to be upregulated in lungs of patients with chronic obstructive pulmonary disease (COPD), and induced release of IL-8 from U937 cells. Conversely, saturated aldehydes had no effect. This indicated that alpha, beta-unsaturated aldehydes such as 4-HNE (an ingredient of cigarette smoke) are likely to be pivotal in activating macrophages that may ultimately result in the destructive inflammatory reaction involved in the course of disease in COPD (8).

TRPA1 is also expressed in cultured human airway cells including epithelial cells, smooth muscle cells, and fibroblasts. *In vitro*, acrolein and cigarette smoke aqueous extract (CSE) (both TRPA1 agonists) induced the release of IL-8 TRPA1 dependently which was reduced by pharmacological TRPA1 blockade. TRPA1 expression was highly co-localized with TRPV1 expression in airway sensory nerves and the activation of both TRPA1 and TRPV1 channels induced the release of the pro-inflammatory neuropeptide SP. Interestingly however, the pro-inflammatory effects of acrolein and CSE were independent of sensory neuronal activation. After 24 h of intra-tracheal instillation with both compounds, neutrophil chemoattractant chemokine (KC) was increased in bronchoalveolar lavage (BAL) independent of pretreatment with a SP receptor/NK1 antagonist. In contrast, intra-tracheal instillation of capsaicin or SP had no effect on KC levels. Furthermore, pretreatment with a TRPA1 antagonist (HC) decreased KC release. Moreover, BAL from TRPA1-deficient mice did not show any release of acrolein- and CSE-induced KC. Thus, KC accumulation-derived inflammation was independent of neurogenic factors, and non-neuronal TRPA1 was shown to be essential in this model of inflammatory airway disorder (9).

In line with these observations of TRPA1 acting as an immune modulator, TRPA1 expression was detected by northern blot, western blot, and immunohistochemical methods in Jurkat T cells as well as in human splenocytes (10). Bertin et al. also confirmed the expression of mouse and human TRPA1 at mRNA and protein level in murine T cells (11).

### TRPM8

TRPM8 is characterized in peripheral sensory neurons as a cold sensor (12, 13). Increasing evidence is accumulating that TRPM8 might also be implicated in inflammatory disorders. Previously, direct evidence for TRPM8 expression in macrophages was reported. TRPM8-like channels could be activated by the TRPM8 agonist icilin measured by the patch-clamp technique in RAW 264.7 macrophages (14). Recently, we observed evidence for TRPM8 expression in several populations of murine macrophages, which modulated inflammatory responses. *In vitro*, TRPM8 activation by menthol induced an anti-inflammatory cytokine profile in murine peritoneal macrophages (increased IL-10 and decreased TNF-α release) (15). Consistently, 1,8-cineol (eucalyptol) and L-menthol (both TRPM8 agonist) were able to inhibit the production of pro-inflammatory cytokines in human monocytes and lymphocytes *in vitro* (16, 17). In our own studies, activation of TRPM8 in wild-type (WT) but not in TRPM8-deficient peritoneal macrophages enhanced phagocytosis of zymosan beads. *In vivo*, phagocytic activity of peritoneal macrophages was impaired in TRPM8-deficient mice compared to WT controls (15).

TRPM8 was also found in human lung epithelial cells. Activation of this channel increased the expression of several cytokines and chemokines, including TNF-α, IL-4, IL-13, IL-1α, and IL-1ß, that modulate the response of other resident cell types in the lung such as immune cells, smooth muscle cells, and sensory neurons (18).

### TRPV1

TRPV1 is expressed in sensory neurons of dorsal root ganglia (DRG), trigeminal, and vagal ganglia (19). Due to its high abundance in nociceptive neurons, TRPV1 acts as a nociceptor marker (20). Most of the TRPV1 expressing DRG neurons co-express peptidergic markers such as SP and CGRP (20). TRPV1 is activated by noxious stimuli such as capsaicin, extracellular acidification, or heat and is sensitized or activated by inflammatory mediators *in vitro* (21–23). Zhao and colleagues found that oxLDL-stimulated bone marrow-derived macrophages showed an increased level of TRPV1 expression and oxLDL activated TRPV1 which led to intracellular Ca^2+^-transients that were abolished by superfusion with the TRPV1 antagonist capsazepine. Capsazepine aggravated the oxLDL-induced lipid accumulation and induced the production of MCP-1 and IL-6 in macrophages. In contrast, pretreatment of bone marrow-derived macrophages with evodiamine or capsaicin (TRPV1 agonists) alleviated lipid accumulation and impaired the production of MCP-1, MIP-2, and IL-6 (24). Capsaicin application to LPS- and IFN-γ-stimulated RAW 264.7 macrophages exhibited inhibitory effects on iNOS and the production of NO, COX-2, and PGE_2_ in a concentration-dependent manner. Capsazepine failed to abolish the effect of capsaicin but rather showed similar inhibitory effects even synergistic with capsaicin on PGE_2_ released from LPS-stimulated peritoneal macrophages (25). In another report, capsaicin failed to modulate the protein/mRNA levels of COX-2, whereas capsazepine exhibited an inhibitory effect on the COX-2 levels produced from LPS-stimulated peritoneal macrophages (25). Both capsaicin and capsazepine decreased iNOS mRNA levels in LPS/IFN-γ-stimulated peritoneal macrophages in a concentration-dependent manner (25). Since iNOS and COX-2 are regulated by transcription factors like nuclear transcription factor kB (NF-kB) (26), it was examined, whether capsaicin or capsazepine regulated the activation of NF-kB. Both, capsaicin and capsazepine, blocked the degradation of IkB-a induced by LPS-stimulated peritoneal macrophages, reflecting that both capsaicin and capsazepine inhibit the activation of NF-kB. Due to the inability of capsazepine to block the effect of capsaicin, the authors suggested that peritoneal macrophages do not express TRPV1 but rather a TRPV1-like protein (25). Intriguingly, these results become more complicated in their interpretation since we were recently able to show that capsazepine is also a potent TRPA1 agonist (2).

In a sepsis model of cecal ligation and puncture, LPS-stimulated TRPV1-deficient peritoneal macrophages showed impaired phagocytosis compared to unstimulated controls (27). LPS/SP co-stimulated TRPV1^−/−^ macrophages were shown to restore phagocytic activity, an effect that was abolished by pretreatment with a selective antagonist of the SP/NK1 receptor. In contrast, CGRP-stimulated TRPV1-deficient macrophages did not show a significant difference in response to LPS (27). Furthermore, a TRPV1 antagonist decreased phagocytosis of LPS-stimulated WT macrophages compared to control cells (27). Recently, a previously unknown role of the endocannabinoid system in regulating immune homeostasis TRPV1 dependently was reported. Activation of TRPV1 by capsaicin induced production of the endocannabinoid anandamide in myeloid cells and promoted the presence of immunosuppressive CXCR1^hi^ macrophages *via* anandamide acting on CB2 receptors expressed in the enteric nervous system. Moreover, this mechanism also provided protection from experimental autoimmune diabetes (28).

Amantini et al. could demonstrate that distinct thymocyte subsets express TRPV1, which is required for capsaicin-induced apoptosis (29). Functional expression of TRPV1 was furthermore shown by Bertin et al. in primary murine CD4^+^ T cells (30). The authors could show that the co-stimulatory molecules CD4 and TRPV1 were co-localized within the plasma cell membrane of CD4^+^ T cells and stimulation with capsaicin triggered calcium ion influx.

In bronchial epithelial cells, TRPV1 was able to control the expression of pro-inflammatory cytokines such as IL-6 and IL-8 due to its modulation of calcium traffic between the intra- and extracellular compartment (31). IL-8 is an essential chemotactic protein produced by neutrophils in the lung (32), which provides the molecular basis for a vital epithelial–immune interaction.

### TRPV4

TRPV4 was originally identified to be involved in the regulation of osmotic homeostasis (33). Furthermore, TRPV4 is activated by mechanical stress and non-noxious heat (34). The prominent role of TRPV4 in visceral nociception was highlighted by recent work (35, 36). Beyond TRPV4 function in neurons, several reports suggest an important physiological function of non-neuronal TRPV4. TRPV4 is expressed in lung and gut epithelial cells and immune cells including macrophages, monocytes, neutrophils, and T cells (4, 37). Functional TRPV4 expression was recently observed in bone marrow-derived macrophages. The selective TRPV4 agonist GSK1016790A increased the intracellular calcium ion concentration in a dose-dependent manner, an effect that was inhibited by TRPV4 siRNA or a pharmacological blocker and completely abolished in TRPV4-deficient bone marrow-derived macrophages. Using fluorometry *in vitro* and quantifying phagocyted particles *in vivo*, the authors also observed impaired phagocytosis after downregulation of TRPV4 with siRNA in LPS-treated bone marrow-derived macrophages (38).

TRPV4 expression was shown in human airway epithelial cell lines (A549, Beas 2B, and NCI-H292) and primary airway epithelial cells. Selective TRPV4 agonists were shown to trigger calcium influx in NCI-H292 and increased the release and secretion of IL-8 and PGE_2_ in a time- and concentration-dependent manner (37). *In vivo*, intranasal administration of the TRPV4 agonist 4α-PDD enhanced the concentration of KC, which subsequently led to the recruitment of neutrophils in BAL fluids in WT but not TRPV4-deficient mice (37), indicating an *indirect* epithelial–immunological interaction. However, a *direct* role for TRPV4 expression in the function of murine alveolar macrophages was also demonstrated. 4α-PDD triggered calcium influx and subsequently production of superoxide and nitric oxide in alveolar macrophages of WT but not TRPV4-deficient mice. Likewise, the adoptive transfer of TRPV4-expressing alveolar macrophages into lungs of TRPV4^−/−^ mice restored hypersusceptibility in a model of ventilator-induced mechanical injury (39).

In line with these pro-inflammatory functions of TRPV4, Majhi and colleagues found endogenous expression of different TRPV isoforms in Jurkat, primary human T cells, and mouse T cells isolated from spleens. Moreover, they observed calcium influx in T cells upon treatment with different TRPV4 agonists. Furthermore, *in vitro* activation of TRPV4 in T cells resulted in upregulation of TRPV1 and TRPV4 channels, T cell proliferation, and production of the pro-inflammatory cytokines IFN-γ, TNF-α, and IL-2. This effect was blocked pharmacologically suggesting that TRPV4-mediated calcium influx might play a crucial role in T cell-mediated immune responses (40).

## TRP Channels in Immune Cells and Epithelia—Role in Models of Intestinal Inflammation

**Figure 1:**
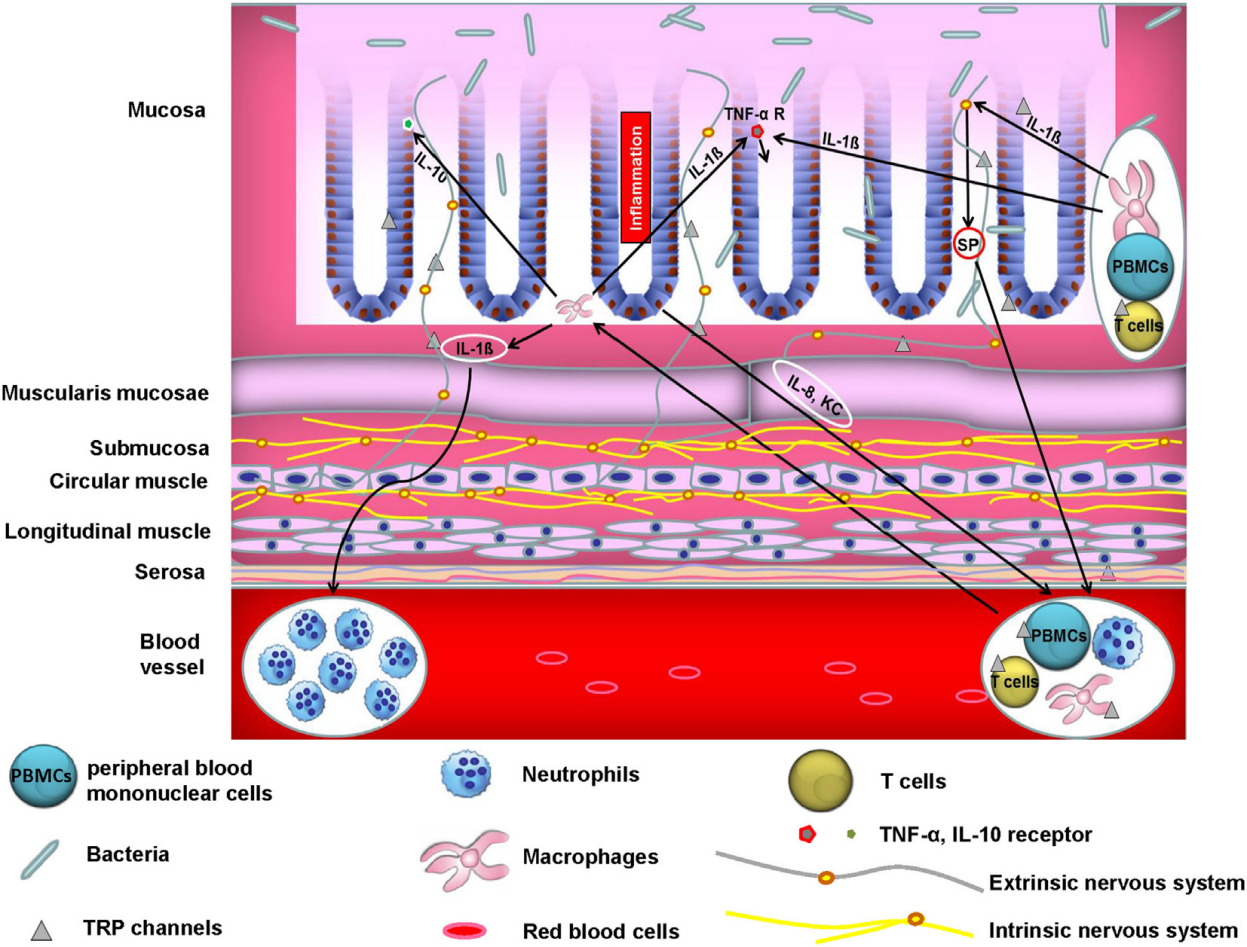
Crosstalk between neurons, immune cells, and epithelial cells controls colonic homeostasis. A vital interplay between neurons, immune cells, and epithelial cells is crucial for colonic homeostasis. Aberrant function of one or more of these players may cause inflammation. For instance, activated macrophages release IL-1ß which among other cytokines acts as a chemoattractant to human peripheral blood mononuclear cells (PBMCs) (45). On the other hand, IL-1ß increases TNF-α receptor expression in epithelial cells which may promote TNF-α-mediated inflammatory responses and subsequently colitis. Moreover, IL-1ß acts on peptidergic sensory neurons through induction of pro-inflammatory substance P (SP) release. SP in turn induces migration of polymorphonuclear leukocytes *in vivo* (46). In addition, epithelial IL-8 and KC induce immune cell infiltration into the colonic wall. IL-10 is an essential immunoregulatory cytokine (47). Epithelial cells from murine small intestine and colon express the IL-10 receptor and its stimulation blocks IFN-γ-mediated pro-inflammatory effects (48). Moreover, not only epithelial cells but also enteric neurons express cytokines and chemokines such as IL-8, whose neuronal production is promoted by IL-1ß *via* MAPK signaling pathways (49).

The majority of published literature ascribes the role of TRP channels in modulation of experimental colitis to its capacity to attenuate neuropeptide release and subsequently neurogenic inflammation. In accordance with this, we have found that TRPA1 and the pro-inflammatory neuropeptide SP in extrinsic primary afferent neurons are fundamental for the development of TNBS colitis. Pharmacological blocking of TRPA1 attenuated chronic colitis through inhibition of neuropeptide release (41). However, the role of extra-neuronally expressed TRP channels in the pathogenesis of intestinal inflammation is emerging and results in a complex crosstalk of different cellular compartments (see Figure 1). A recent study stressed the important role of extra-neuronal TRPA1 and TRPV1 receptor expression in this context. TRPA1 and TRPV1 were expressed in macrophages and epithelial cells in both healthy and inflamed human and murine colons (42). Since macrophages are major producers of TNF-α and activation of TRPA1 was able to impair the expression of TNF-α in distal colon homogenates during colitis, it is likely that TRPA1 in macrophages mediated the anti-colitogenic effect. In addition, employing different mouse models of colitis including IL-10 knockout mice as well as adoptive T cell transfer models of colitis, it was shown that the genetic deletion of TRPA1 in CD4^+^ T cells caused intestinal inflammation *via* induction of the transcription factor Tbet which subsequently increased production of the pro-inflammatory cytokines IFN-γ and IL-2 (11) and resulted in a higher capacity to differentiate into TH1 effector cells. Additionally, CD4^+^ T cells isolated from TRPV1 knockout mice showed impaired calcium influx upon T cell receptor stimulation, resulting in the inactivation of the transcription factors NFAT and NFkB. Likewise, TRPV1-deficient CD4^+^ T cells failed to induce colitis in transfer models of colitis as TRPV1-deficient CD4^+^ T cells showed decreased production of the pro-inflammatory cytokines IFN-γ, TNF-α, and IL-17 (30). An important extra-neuronal role of TRPV4 was recently demonstrated with regard to colonic inflammation. TRPV4 mRNA was found to be upregulated in colonic intestinal epithelial cells from mice with dextran sulfate sodium (DSS)-induced colitis. TRPV4 activation led to chemokine and cytokine release such as IL-8, IP-10, MIG, and MCP-1, which indicated a potential role of TRPV4 in activating pro-inflammatory signaling pathways that might induce the recruitment of macrophages and other immune cells. In addition, intrarectal enemas with the TRPV4 agonist 4α-PDD induced acute and chronic colonic inflammation in mice (4). Finally, we could recently show that mice that were reconstituted with TRPM8-deficient macrophages exhibited increased susceptibility to DSS, demonstrating a fundamental role of constitutive TRPM8 expression in macrophages in the context of colitis (15). Two recent reports, however, favor a dominant role for TRPM8 expression in controlling neuropeptide release and, thus, colitis development (43, 44). TRPM8 was shown to modulate the release of CGRP in the colonic microenvironment from peptidergic sensory neurons, which might have directed the protective effects of CGRP on CD11^+^ dendritic cells (44).

## Conclusion

The role of TRP channels reaches beyond the control of immunomodulatory neuropeptide release from sensory nerve endings. Many TRP channels are expressed in various immune cells, especially in macrophages and T cells. Here, they modulate many functions such as cytokine expression and release, migration, or phagocytic activity. Moreover, in a third compartment, the epithelial layer, TRP channel expression was also found to be relevant in the pathogenesis of many inflammatory disorders mainly through controlling chemokine/cytokine expression and release. Thus, a vital interplay between neurons, epithelia, and mucosal immune cells seems to maintain homeostasis in different organs, for example, the gut, the lung, and the vascular system and disruption of one or more of these players may induce disease (see Figure 1). Thus, targeting TRP channels and neuropeptide receptors might represent a promising new therapeutic approach in various inflammatory disorders such as inflammatory bowel disease, asthma, COPD, and atherosclerosis.

## Author Contributions

MK: conception and writing of the manuscript; KA: conception and writing of the manuscript; CW and CY: conception and writing of the manuscript; SW and CB: conception of the manuscript, revising it critically for important intellectual content, and final approval of the version to be published; ME: conception and writing of the manuscript, revising it critically for important intellectual content and final approval of the version to be published.

## Conflict of Interest Statement

The authors declare that the research was conducted in the absence of any commercial or financial relationships that could be construed as a potential conflict of interest.

## Supplementary Material

Table S1 Expression and biological effects of the different TRP channels in immune cells and epithelia.
