# Supplementary material for: Functional Role of Transient Receptor Potential Channels in Immune Cells and Epithelia
Source: Front Immunol. 2018 Feb 7;9:174. doi: 10.3389/fimmu.2018.00174 (PMC5808302; doi:10.3389/fimmu.2018.00174)
Supplement: Table S1 — Expression and biological effects of the different TRP channels in immune cells and epithelia. [file Table_1.PDF]

| TRP channels | Expression in epithelial cells and immune cells | Functional role of TRP activation |
|---|---|---|
| **TRPA 1** | • Murine macrophages (7)<br>• Murine colonic lamina propria lymphocytes (11)<br>• Human T cells (10)<br>• Human and murine airway epithelial cells (9) | • Regulation of pro-inflammatory cytokines in macrophage foam cells (6)<br>• ⇧ Release of IL-8 and KC levels in murine BAL fluid (9)<br>• ⇧ Production of the pro-inflammatory cytokines IFN-γ and IL-2 in colonic lamina propria lymphocytes (11)<br>• Induction of TNBS colitis (41) |
| **TRPM8** | • Murine macrophages and macrophage cell lines (14,15)<br>• Human monocytes and lymphocytes (16,17)<br>• Human lung epithelial cells (18)<br>• Inflamed colonic tissues (DSS colitis, patients with CD) (43,44) | • Induction of an anti-inflammatory cytokine profile (⇧ IL-10, ⇩ TNF-α) in macrophages<br>• ⇧ Macrophage phagocytosis<br>• ⇩ Production of pro-inflammatory cytokines in human monocytes and lymphocytes in vitro (16,17)<br>• Menthol (TRPM8 agonist) enemas were protective in mice with DSS colitis<br>• TRPM8-deficient mice exhibited ⇧ susceptibility of DSS colitis (15)<br>• ⇧ Expression of TNF-α, IL-4, IL-13, IL-1α and IL1-ß in human lung epithelia (18) |
| **TRPV1** | • Murine macrophages (24,25)<br>• Human and murine $CD4^+$ T cells (11,40)<br>• Human airway epithelial cells (31,32) | • ⇩ lipid accumulation and ⇩ production of MCP-1, MIP-2 and IL-6 in macrophages in the context of atherosclerosis (24)<br>• ⇩ production of iNOS, NO, COX-2 and PGE2 in murine peritoneal macrophages (25)<br>• Up-regulation of TRPV1 and ⇧ release of pro-inflammatory cytokines such as IFN-γ, TNF-α and IL-2 might play a crucial role in T cell mediated immune responses and T cell proliferation (40)<br>• Regulatory role in IL-6 and IL-8 expression in human airway epithelial cells (31,32) |
| **TRPV4** | • Macrophages, monocytes, neutrophils, T cells, bone marrow-derived macrophages (BMDMs) (4,37,38,40).<br>• Human airway epithelial cell lines (A549, Beas 2B, and NCI-H292) (39)<br>• Lung and gut primary epithelial cells of mice and men (4,37, 40) | • ⇧ Phagocytosis of bone marrow-derived macrophages (38)<br>• ⇧ Production of superoxide and nitric oxide in alveolar macrophages (37)<br>• ⇧ Release of IL-8 and PGE2, KC and ⇧ recruitment of neutrophils in BAL fluids (37)<br>• Adoptive transfer of TRPV4 expressing alveolar macrophages into lungs of TRPV4-/- mice restored hyper-susceptibility in a model of ventilator-induced mechanical injury (39)<br>• Up-regulation of TRPV4 and ⇧ release of pro-inflammatory cytokines such as IFN-γ, TNF-α and IL-2 might play a crucial role in T cell mediated immune responses and T cell proliferation (40)<br>• ⇧ Chemokine and cytokine release such as IL-8, IP-10, MIG, and MCP-1 from intestinal epithelial cells induced acute and chronic colonic inflammation in mice (4) |
